# Citrin Deficiency: Biochemical Mechanisms Underlying a Complex Hepatic Metabolic Disorder

**DOI:** 10.3390/biom16101460

**Published:** 2026-10-07

**Authors:** Ismael García-Lobo, Araceli del Arco, Jorgina Satrústegui, Laura Contreras

**Affiliations:** 1 Departamento de Biología Molecular, Universidad Autónoma de Madrid, 28049 Madrid, Spain; ismael.garcia@cbm.csic.es (I.G.-L.); jsatrustegui@cbm.csic.es (J.S.); 2 Instituto Universitario de Biología Molecular (IUBM), Centro de Biología Molecular Severo Ochoa, Universidad Autónoma de Madrid, 28049 Madrid, Spain; araceli.arco@uclm.es; 3 Instituto de Investigación Sanitaria Fundación Jiménez Díaz (IIS-FJD), Universidad Autónoma de Madrid, 28040 Madrid, Spain; 4 Facultad de Ciencias Ambientales y Bioquímica, Universidad de Castilla la Mancha, 45071 Toledo, Spain; 5 Centro Regional de Investigaciones Biomédicas and Unidad Asociada de Biomedicina, UCLM-CSIC, Universidad de Castilla la Mancha, 45071 Toledo, Spain

**Keywords:** aspartate/glutamate carrier (AGC), Citrin Deficiency (CD), malate–aspartate shuttle (MAS), NADH/NAD^+^ ratio, hepatic metabolism, urea cycle, gluconeogenesis, galactosemia

## Abstract

Citrin Deficiency (CD), caused by pathogenic variants in *SLC25A13*, is a rare inherited metabolic disorder with age-dependent manifestations ranging from neonatal intrahepatic cholestasis to adult-onset citrullinemia. Although the genetic basis of CD is well established, the mechanisms linking loss of the mitochondrial aspartate/glutamate carrier (citrin) to the CD phenotype remain incompletely understood. This review examines current evidence from patients and available cellular and animal models supporting the biochemical mechanisms underlying CD. We discuss how disruption of the malate–aspartate shuttle alters cytosolic redox homeostasis and its consequences for interconnected pathways of carbohydrate, lipid, and nitrogen metabolism, including glycolysis, gluconeogenesis, galactose metabolism, fatty acid oxidation, lipogenesis, and ureagenesis. We also distinguish mechanisms supported by experimental evidence from those that remain hypothetical and highlight major unresolved questions regarding metabolic adaptation, disease progression, and the transition to the adult phenotype.

## 1. Introduction

Citrin Deficiency (CD) is a rare pan-ethnic metabolic disorder with higher prevalence in Asia caused by mutations in the *SLC25A13* gene [1,2,3], coding for citrin/AGC2. Initially identified as a secondary urea cycle disorder associated with low hepatic argininosuccinate synthetase (ASS1) activity [4], the disease was first described as adult-onset Citrullinemia type II (CTLN2), currently renamed Adolescent and Adult Citrin Deficiency (AACD) [5]. Patients show marked elevation of citrulline and ammonium in plasma, with neurological symptoms (disorientation, drowsiness, memory loss, flapping tremor, seizures) that can lead to death from brain edema. Fatty liver, hepatocarcinoma and pancreatitis are frequent complications [1,3]. Later, the disorder was also recognized in newborns as Neonatal Intrahepatic Cholestasis caused by Citrin Deficiency (NICCD) [6], characterized by hypoglycemia, galactosemia, cholestasis, fatty liver and elevated plasma citrulline. NICCD usually resolves during the first year of life with appropriate dietary management, after which patients generally enter a compensated stage with limited clinical manifestations. Nevertheless, a characteristic preference for protein- and lipid-rich foods over carbohydrates is consistently reported [1,7], together with fatigue and symptoms triggered by carbohydrate intake affecting the patient’s quality of life [8]. Besides, a subset of patients develop Failure to Thrive and Dyslipidemia caused by Citrin Deficiency (FTTDCD), characterized by impaired growth, hypoglycemic episodes, and fatty liver [1].

CD has an estimated prevalence of approximately 1:17,000 births in Asia, whereas the adult condition is estimated to affect around 1:100,000 individuals [1,9,10]. The mechanisms underlying this discrepancy in the prevalence remain unknown, as does the shift toward higher penetrance in males during the adult stage, while no sex bias has been reported for NICCD [1,5,9]. More than 140 pathogenic mutations have been identified throughout the *SLC25A13* sequence, affecting localization, dimerization, and transport activity of the protein [11,12]. Although the clinical impact of different mutations has been explored [10], no clear genotype–phenotype correlation has been established.

Citrin belongs to the subfamily of mitochondrial aspartate/glutamate carriers (AGCs), transporters located in the inner mitochondrial membrane that mediate the electrogenic efflux of matrix aspartate in exchange for cytosolic glutamate plus H^+^ [13]. Mammals express two AGC isoforms: aralar, predominantly expressed in brain and skeletal muscle, and citrin, mainly expressed in liver [2,14,15,16]. Aspartate export is required to sustain cytosolic biosynthetic pathways involved in protein and nucleotide synthesis, which is important for cell growth [17]. In addition, aspartate is necessary for tissue-specific metabolic functions, including glutamine and N-acetyl-aspartate synthesis in the brain [18] and to fuel the urea cycle in the liver [4,13,16].

Beyond aspartate transport, AGCs are essential components of the mitochondrial malate–aspartate shuttle (MAS), the major cellular system responsible for transferring cytosolic reducing equivalents into mitochondria [19], as the inner mitochondrial membrane is impermeable to NADH. MAS is formed by AGCs, the oxoglutarate carrier, and the coordinated activity of malate dehydrogenase and aspartate aminotransferase in the cytosolic and mitochondrial compartments. Through the interconversion of malate/oxaloacetate (OAA) and glutamate/aspartate pairs, MAS regenerates cytosolic NAD^+^ while transferring reducing equivalents to the mitochondrial matrix (Figure 1A). Because the NADH/NAD^+^ ratio regulates numerous intermediate metabolic reactions, disruption of MAS activity is expected to broadly affect cellular metabolism. Consistently, pathogenic variants in several MAS components have been associated with severe human diseases [18,20,21], including CD.

Current understanding of Citrin Deficiency (CD) is based on clinical data from patients and experimental studies using animal and cellular models (Table 1). These experimental systems have provided important insights into the metabolic consequences of citrin loss, but each presents specific limitations that should be considered when interpreting the evidence [22]. The single *Slc25a13* knockout (citrin KO) mouse exhibits a relatively mild phenotype, likely due to compensation by the active glycerol-phosphate shuttle in mouse liver [23] and the non-negligible presence of the isoform aralar in mouse livers [16]. Eliminating *Gpd2* enhances the metabolic phenotype, and the double knockout (citrin/gpd2 KO) mouse model shows phenotypic traits consistent with CD [24], but also disrupts global Gpd2 functions [25]. Therefore, findings from this model might not be dependent exclusively on Citrin Deficiency. Similarly, transformed HepG2 cells do not fully reproduce the differentiation and metabolic specialization of primary hepatocytes, whereas patient-derived hepatocyte-like cells provide a complementary but still simplified human model [22,26,27,28]. Accordingly, findings reproduced across independent models and supported by clinical observations are considered more robust, whereas model-specific findings should be interpreted with caution.

The central role of MAS in maintaining cellular redox balance places citrin at the intersection of carbohydrate, lipid, and amino acid metabolism, particularly in the liver. In this narrative review, we provide a biochemical perspective on the metabolic alterations associated with CD, first introducing the relevant metabolic pathways and then discussing the proposed mechanisms underlying their dysregulation and their relationship to the clinical and metabolic phenotype. We integrate evidence from patients and available cellular and mouse models to distinguish mechanisms supported by experimental data from hypotheses inferred primarily from indirect clinical and biochemical observations (Table 1 and Table 2). Finally, we highlight major gaps in current knowledge and discuss future directions needed to advance our understanding of CD pathophysiology.

## 2. Impaired Carbohydrate Metabolic Pathways in CD

### 2.1. Evidence for Impaired Glycolytic Flux in CD

Glycolysis, composed of ten cytosolic enzymes, is the central metabolic pathway for energy production from carbohydrates [58]. The product of glycolysis is pyruvate, with net production of 2 ATP per glucose molecule. Pyruvate can be reduced in the cytosol to produce lactate for NAD^+^ regeneration, but this limits energy production, and pyruvate is preferentially taken up by the mitochondrial pyruvate carrier into the mitochondrial matrix for complete oxidation via pyruvate dehydrogenase and the tricarboxylic acid (TCA) cycle. Both activities will generate NADH in the mitochondrial matrix to feed into the electron transport chain for ATP generation (oxidative phosphorylation, OXPHOS), with a final yield of approximately 30–32 ATP per glucose molecule. Alternatively, pyruvate can be transformed to lactate with NAD^+^ regeneration, although this route is less energetically efficient. Therefore, glycolysis is a cornerstone for the utilization of carbohydrates for energy production in all cells.

Flux regulation occurs at three irreversible steps catalyzed by hexokinase, phosphofructokinase-1, and pyruvate kinase [58,59]. In addition, glyceraldehyde-3-phosphate dehydrogenase (GAPDH) catalyzes the only redox-dependent step of the pathway and requires NAD^+^ as a cofactor, making it a critical bottleneck when the NADH/NAD^+^ ratio is elevated [60], as occurs during alcohol metabolism [61]. Therefore, glycolytic production of pyruvate to support mitochondrial OXPHOS depends on the capacity of the redox shuttles to reoxidize NAD^+^ [61] and avoid reductive stress (elevated NADH/NAD^+^) (Figure 1B). Evidence for this role of MAS has been demonstrated in several systems. Knocking out aralar or chemically inhibiting MAS limits glycolytic pyruvate utilization by mitochondria and reduces OXPHOS from glucose in synaptosomes, cultured neurons and in the brain in situ [62,63,64]. Deletion of *SLC25A13* in HepG2 cells creates a partial restriction in glycolytic flux, observed as accumulation of metabolites upstream of GAPDH and depletion of downstream glycolytic intermediates [26], which is consistent with the elevation in the NADH/NAD^+^ ratio. This increase was confirmed in vitro using cultured primary hepatocytes from citrin KO mice [16], and it is inferred as an elevated lactate/pyruvate ratio measured in liver perfusion experiments or hepatic extracts from CD mouse models [23,24]. In patients, redox imbalance is inferred from the elevation in the lactate/pyruvate ratio in plasma analysis [29,30,31]. Although it is not a critical trait across the different manifestations [1], the lactate/pyruvate ratio may be used in differential diagnosis in the compensatory stage [65]. Table 1 summarizes the evidence of impaired glycolysis across patients and cellular and mouse CD models. The metabolic consequences of glycolysis disruption due to an elevated NADH/NAD^+^ ratio vary. First, carbon flux through the TCA might become disrupted by inefficient pyruvate supply to mitochondria, lowering mitochondrial respiration and ATP production [26]. TCA metabolites were decreased in *SLC25A13* KO HepG2A cells [26], while hepatic malate, citrate and α-ketoglutarate (αKG) were elevated in CD mouse models, but levels were reduced by sucrose administration to mice [33], indicating differences in carbon supply to TCA in CD models. Patients also show increased plasma citrate and αKG, but conversely decreased malate, fumarate and succinate, suggesting a more profound alteration of the cycle that was not reversed by pyruvate administration [29]. These data suggest a possible imbalance between anaplerotic and cataplerotic mechanisms that remain unexplored.

Second, it precludes the use of glycerol or dextrose infusions to treat brain edema caused by hyperammonemic crisis in CD patients [5,9]. Glycerol, which is used as an osmotic agent to reduce edema, is metabolized to glycerol-3-phosphate with ATP cost and then converted to dihydroxyacetone phosphate for oxidation or glucose synthesis, a reaction that requires NAD^+^. In CD patients, these treatments will impose an additional energetic and redox stress on the hepatocytes and even cause death to the patient.

Third, it has been proposed as the molecular basis for the peculiar aversion of patients towards alcohol and carbohydrate-rich foods, shifting their diet towards increased protein and lipid intake [7,32], a characteristic phenotypic trait also observed in mouse models [35]. This dislike is not due to a lack of craving for sweetness [35], and has been proposed to result, at least in part, from hepatic intolerance to carbohydrate intake. This intolerance is supported by hyperammonemia induced by oral administration of sucrose to CD mouse models [24,33], but its metabolic basis remains incompletely understood. In patients, however, the relationship between carbohydrate consumption and symptoms is based primarily on dietary preference, clinical observations and patient-reported outcomes, such as adverse symptoms associated with carbohydrate intake and chronic fatigue [8,31].

### 2.2. Galactosemia as an Early Manifestation of CD

Galactose is produced from lactose, a key disaccharide of milk, the main nutrient source during the neonatal period. After hydrolysis by intestinal lactase, galactose enters central metabolism for energy production via the Leloir pathway mainly in the liver [66]. This pathway consists of four reactions catalyzed by galactose mutarotase (GALM, EC 5.1.3.3), Galactose kinase (GALK, EC 2.7.1.6), galactose-1-phosphate uridylyltransferase (GALT, EC 2.7.7.12) and UDP-glucose 4′-epimerase (GALE, EC 5.1.3.2) (Figure 1C). Mutations in the Leloir enzymes cause galactosemia [67,68], a life-threatening condition clinically suspected when there is increased galactose in plasma. However, galactosemia might be secondary to congenital hepatic vascular anomalies or liver diseases.

From a biochemical mechanistic perspective, MAS deficiency may impair galactose metabolism through two non-exclusive mechanisms. First, the catalytic mechanism of GALE requires a tightly bound NAD^+^ cofactor that transiently oxidizes the sugar as part of the epimerization mechanism [68]. NADH was reported to competitively inhibit GALE activity in vitro, with approximately 50% inhibition reported at an NADH/NAD^+^ ratio of 1:20 in bovine enzymes [56]. Second, galactose metabolism requires an initial ATP-dependent phosphorylation step catalyzed by GALK. Therefore, the reduced glycolytic flux and mitochondrial energy metabolism associated with CD (see Section 2.1) could potentially limit GALK activity due to decreased ATP availability. However, these two hypothetical mechanisms have not been directly demonstrated in CD models.

NICCD patients present with galactosemia, and it is considered a key marker of this stage of CD. In addition to galactose, other intermediates of the Leloir pathway, such as galactitol, can accumulate and can be detected in urine samples from NICCD patients [40,69]. Galactose intermediates are established toxic metabolites in primary galactosemia and have been associated with complications including hepatotoxicity and cataracts [6,38,39]. Following the rationale established in primary galactosemias, lactose-free formula to restrict metabolic exposure to galactose and accumulation of toxic intermediates is usually employed as therapy in NICCD [36,37]. Despite the clinical relevance of galactosemia in NICCD, no direct experimental analysis of galactose metabolic flux has yet been performed in currently available CD models to determine whether NICCD-associated galactosemia primarily reflects impaired galactose metabolism, secondary hepatic dysfunction, or a combination of both. Most in vivo studies rely on adult mice that no longer consume milk [23,24], and galactose metabolism has not been systematically addressed in existing cellular models [26,27,28]. Consequently, the hypothetical redox and energy-dependent mechanisms of CD galactosemia remain experimentally unvalidated. Nonetheless, the early and consistent manifestation of galactosemia in NICCD raises the possibility that galactose metabolism might represent a physiological redox stress test for the neonatal liver. This concept, which remains hypothetical and requires experimental validation, might be useful for revealing the vulnerability of CD hepatocytes under conditions of obligatory carbohydrate utilization.

### 2.3. Citrin-MAS Dysfunction Affects Hepatic De Novo Glucose Synthesis

Hypoglycemia is a recurrent clinical feature of CD present even in the compensated period following neonatal cholestasis, although it is less frequently observed in AACD [1,5,42,43]. In clinical settings, hypoglycemia is most pronounced during reduced food intake or metabolic stress [43] and often resolves with appropriate dietary management, indicating a functional rather than intrinsic impairment of hepatic glucose production. These observations point to altered gluconeogenic capacity as a contributing factor to glucose dysregulation in CD.

A major function of the liver is to balance glucose in the organism, especially during fasting periods. Accordingly, glucose is produced by glycogenolysis of previously stored glycogen, but when stores are depleted, de novo synthesis from different precursors takes place. Gluconeogenesis (GNG, Figure 1D) may occur in the postprandial state if enhanced glucose flux is required, for example by exercise. Furthermore, tracer studies demonstrate that de novo glucose-6-phosphate production through the GNG route is an important contributor to glycogenogenesis [70]. Thus, GNG is not restricted to the absence of food, but it is required for glycogen stores and baseline glycemia maintenance throughout the day.

GNG, located mainly in the periportal area of the liver, uses different precursors to sustain blood glucose levels after glycogen stores are depleted. The main glucose precursors are lactate (7–18%), glycerol (3–7%), alanine (8–11%) and glutamine (5–8%), with variable contributions dependent on the nutrient status, energy requirement and metabolic context [71]. Glycerol and lactate utilization depend on the cytosolic NADH/NAD^+^ ratio to enter the GNG pathway (Figure 1D). Lactate is oxidized by cytosolic lactate dehydrogenase, which uses NAD^+^ as a cofactor to form pyruvate and NADH. Glycerol is phosphorylated by glycerol kinase (GK) to produce glycerol 3-phosphate (G3P), a substrate of glycerol-3-phosphate dehydrogenase 1 (GPD1), a cytosolic NAD^+^/NADH-dependent enzyme that catalyzes its conversion to dihydroxyacetone phosphate (DHAP). Consequently, glycerol enters the gluconeogenic pathway as DHAP, bypassing the complex mitochondrial steps required for lactate. An increase in the cytosolic NADH/NAD^+^ ratio would thermodynamically oppose the oxidation of lactate to pyruvate and the conversion of G3P to DHAP, thereby potentially limiting glucose production from both precursors, although at distinct steps of the pathway. This situation was studied in rodents after ethanol administration [72] or in the presence of aminooxyacetate, which inhibits the transamination steps of MAS [73]. Conversely, decreasing the NADH/NAD^+^ ratio by *Lb*NOX augments GNG from glycerol and lactate in vivo [74]. Consistently, GNG from lactate has been reported to be reduced in citrin KO liver perfusion experiments while normal glucose output was obtained with pyruvate, a substrate independent of NADH/NAD^+^ ratio [23]. These results align with the hypoglycemia observed in vivo in the citrin KO (fasting condition) and citrin/gpd2 KO (both in fed and fasting conditions) mouse models [24], reflecting the hypoglycemia reported in patients.

Non-reduced precursors might also require citrin activity for OAA efflux from mitochondria in the form of aspartate [75]. OAA is generated in the matrix by action of pyruvate carboxylase or in the TCA cycle, and since OAA cannot cross the mitochondrial membrane directly, it must be exported as malate or aspartate [75]. Interestingly, liver-specific deletion of the mitochondrial dicarboxylate carrier (DiC/SLC25A10) causes mild hypoglycemia while retaining a certain level of glucose output, indicating that another efflux pathway for an OAA derivative exists [57]. The authors found enriched aspartate from ^13^C-labeled lactate or pyruvate as precursors [57], which points to AGCs as the alternative route. This mechanism may extend to GNG from amino acids, which are considered largely redox-independent substrates (Figure 1D). Interestingly, the serum profile in NICCD patients shows decreased levels of relevant gluconeogenic amino acids [50,76,77], although whether this reflects increased utilization for hepatic glucose production or other mechanisms remains unclear. Notably, the contribution of amino acids to hepatic glucose production in CD has not been systematically addressed, representing an important gap in knowledge, particularly in light of the marked preference for protein-rich foods of patients [7], despite the predicted impairment of amino acid catabolism due to urea cycle dysfunction (see Section 4).

## 3. Disrupted Lipid Metabolism in CD

Hepatic steatosis is a frequent finding in CD patients [1,5,9] indicating altered lipid metabolism, which is also reproduced in cellular [26,27] and animal models [24]. Steatosis arises from accumulation of triglycerides (TG) exceeding 5%, while healthy livers contain 1–2% TG by weight [78,79,80]. The liver, a key regulator of systemic lipid metabolism, integrates nutrient intake with nutritional requirements in peripheral tissues exporting TG as very-low-density lipoproteins (VLDL), with a constant flux of fatty acids (FA) towards the liver from adipose tissue TG hydrolysis [78]. FA entering hepatocytes are partitioned for oxidation (energy production) or esterification for the production of TG and other lipids [78] depending on physiological and nutritional state, with a fine balance between generation and disposal of FA. We will focus on the two major pathways contributing to hepatic TG balance (FA synthesis and oxidation), although contributions from other factors (FA uptake, adipose lipolysis, VLDL synthesis and export) cannot be excluded. MAS does not directly participate in either de novo lipogenesis (DNL) or fatty acid oxidation (FAO). Therefore, understanding steatosis in CD requires consideration of indirect metabolic consequences rather than direct enzymatic blockade. To frame this discussion, we briefly summarize the core biochemical features of DNL and FAO, in relation to CD metabolic effects.

### 3.1. Potential Mechanisms Linking MAS Dysfunction to Lipogenesis

DNL is the cytosolic pathway for new FA synthesis from non-lipid precursors (carbohydrates, amino acids), triggered by excess nutrients. The first step is the energy-dependent carboxylation of acetyl-CoA to produce malonyl-CoA (Figure 2A), which binds to fatty acid synthase (FASN) to elongate a growing acyl chain with NADPH expenditure, finally releasing palmitoyl-CoA, which is used for complex lipid synthesis [59,81]. Consequently, DNL is highly dependent on cellular energetic and redox state, requiring sustained ATP production, cytosolic reducing power (NADPH), and continuous provision of acetyl-CoA. The major cytosolic source of cytosolic NADPH is the oxidative pentose phosphate pathway (PPP); other cytosolic enzymes that produce NADPH include the malic enzyme (ME1), isocitrate dehydrogenase 1 (IDH1), and dehydrogenases involved in folate metabolism.

An early hypothesis to explain steatosis in CD proposed the upregulation of the citrate shuttle to compensate for the citrin-MAS defect [82]. In the citrate shuttle [83], mitochondrial citrate export and subsequent cleavage generate cytosolic acetyl-CoA for lipogenesis and OAA. This OAA can be recycled back to mitochondria as malate (with NAD^+^ generation by MDH1) or as pyruvate after further conversion of malate by ME1 with generation of NADPH, which can be used for lipid synthesis. Alternatively, increased cytosolic OAA availability could, in principle, support cytosolic aspartate production through GOT1 converting OAA and glutamate to aspartate and α-ketoglutarate (Figure 2A), in the reverse direction of the GOT1 reaction operating in the MAS. Therefore, this pathway could provide an alternative route for cytosolic redox balancing and aspartate production. However, if operative, this compensatory pathway could increase the availability of cytosolic acetyl-CoA and NADPH for lipogenesis, although increased net DNL flux has not been directly demonstrated in CD.

In favor of this hypothesis, Yau et al. [26] reported molecular changes consistent with increased lipogenic activity in a cellular model for CD, such as reduced phosphorylation of acetyl-CoA carboxylase and increased FASN [26]. Transcriptomic analysis of citrin/gpd2 KO livers showed elevated *Acly*, together with other mRNA encoding proteins involved in lipid synthesis/uptake (*Cd36*, *Vldlr*, *Fads3*, *Elov 6*) [24] (Table 1). However, direct flux-based evidence demonstrating increased citrate export or enhanced DNL activity in CD models is currently lacking, and the source of citrate to sustain the hypothetical flux towards DNL remains unclear. An interesting possible source of citrate, yet untested, is glutamine. Reductive carboxylation of glutamine has been reported for mitochondrial-defective cells [84,85] and can generate cytosolic citrate for lipogenesis independently of glycolysis as an alternative pathway in liver [86]. This hypothesis may be particularly relevant in CD, as patients exhibit a preference for protein-rich diets, and recent studies have linked high protein intake to increased hepatic lipogenesis [87]. However, whether this dietary feature contributes to hepatic steatosis in CD remains unknown, and the proposed contribution of glutamine-derived citrate to lipogenesis remains speculative. Importantly, increased availability or expression of lipogenic components does not by itself establish increased DNL flux. Likewise, increased citrate shuttle activity and the contribution of glutamine to lipogenesis should currently be considered hypothetical. Direct experimental validation through dietary interventions combined with isotope-resolved metabolic flux analysis will be required to determine the relative contribution of different substrates to lipogenesis in this context.

### 3.2. Fatty Acid Oxidation and CD

A major pathway counterbalancing hepatic lipid synthesis is fatty acid oxidation (FAO), mainly located in mitochondria [78,79]. Hepatic FAO requires the entry of FA into the mitochondrial matrix, where they undergo progressive degradation to generate acetyl-CoA and reducing equivalents that support oxidative phosphorylation (Figure 2A). To enter the pathway, free FA must first be activated to acyl-CoA by FA thiokinases, with ATP expenditure. Therefore, although the overall balance is elevated ATP production, FAO requires an initial investment of energy [59,78], rendering it sensitive to alterations in mitochondrial function.

Early biochemical research proposed reciprocal inhibition between MAS and FAO [88,89]. Both pathways contribute to reducing equivalents to mitochondrial oxidative metabolism, although FAO generates NADH and FADH_2_ within the mitochondria, whereas MAS transfers cytosolic reducing equivalents to the mitochondrial compartment. Their relative activity may therefore depend on the capacity of the respiratory chain to oxidize NADH and maintain mitochondrial redox balance. From this perspective, impaired MAS in CD could theoretically favor FAO as an alternative source of reducing equivalents and energy. However, experimental evidence does not support this prediction.

First, decreased FAO was determined in a cellular model for CD, together with reduced expression of peroxisome proliferator-activated receptor alpha (PPARα), a key transcriptional regulator of FAO genes and an altered acylcarnitine profile [26]. Pharmacological activation of PPARα partially rescues lipid accumulation in vitro in another cell model for CD [27], supporting a functional role for impaired FAO in the steatosis observed in vivo (Table 1 and Table 2).

Second, the dysregulation of PPARα is not consistently reported in mouse models [24,34]. Nevertheless, the proposed impairment of FAO is consistent with the increased plasma FA levels observed in these models [24], although other factors, such as altered adipose lipolysis or fatty acid uptake, may also contribute. Clinical data, particularly in AACD patients, indicate that decreased expression of PPARα and FAO-related enzymes correlates with steatosis severity [44], providing additional evidence of altered lipid handling. Additionally, altered plasma profiles of carnitine and acylcarnitines, especially an increase in C2-carnitine and long-chain acylcarnitines, have been reported in patients [45,76]. Although these metabolites are considered biomarkers of mitochondrial disorders affecting FAO [90], they are not specific for CD and do not by themselves establish impaired mitochondrial FAO; however, they might have potential diagnostic utility when combined with other clinical findings [46]. Since in vivo hepatic FAO flux has not been directly determined in CD, its contribution to steatosis remains uncertain.

Third, a key functional consequence of impaired FAO is reduced ketogenesis. During fasting, hepatic FAO provides acetyl-CoA for the synthesis of ketone bodies, acetoacetate and β-hydroxybutyrate, which serve as alternative energy substrates for extrahepatic tissues, including the brain. In CD, both ketotic and hypoketotic hypoglycemia have been reported [29,47,48], but overall evidence suggests altered FA handling. This suggests a defect in mitochondrial FA processing and may contribute to the energetic imbalance observed in CD. Interestingly, medium-chain triglycerides (MCTs) are beneficial in CD patients and are included in dietary management [91]. This suggests that medium-chain FA can be efficiently utilized as an energy source, making their benefit appear paradoxical in the context of the proposed impaired FAO in CD. This observation suggests that at least some aspects of hepatic FA utilization remain functional.

### 3.3. Alterations in Lipid Metabolism Regulation in CD

DNL and FAO are reciprocally modulated by metabolic intermediates and transcriptional programs. Key metabolites such as acetyl-CoA, citrate, and malonyl-CoA exert allosteric regulation on rate-limiting enzymes: citrate activates acetyl-CoA carboxylase (ACC), promoting lipogenesis, whereas malonyl-CoA inhibits carnitine palmitoyltransferase 1 (CPT1), thereby restricting mitochondrial FAO. These mechanisms ensure a tight balance between lipid synthesis and degradation. Besides, DNL critically depends on cytosolic NADPH availability from the pentose phosphate pathway (PPP) and malic enzyme activities. Thus, carbohydrate metabolism contributes both carbon and reducing power for lipogenesis.

At the transcriptional level, lipid metabolism is mainly controlled by three transcription factors, SREBP-1, ChREBP and PPARα (Figure 2B). Sterol-responsive element-binding protein (SREBP-1), activated by insulin, promotes expression of lipogenic genes such as ACC and FASN, while carbohydrate-responsive element-binding protein (ChREBP) responds to glucose-derived metabolites to regulate glycolytic and lipogenic pathways [34,92]. In contrast, PPARα drives fatty acid oxidation by inducing genes involved in mitochondrial and peroxisomal FAO [93]. SREBP-1 signaling is not known to be affected in CD. PPARα is downregulated by a non-completely understood mechanism, involving increased c-Jun N-terminal kinase (JNK) activity, as reported in AACD patients with consequent reduced expression of enzymes involved in FAO [44]. A potential link between CD-associated redox alterations and lipogenic transcription is the recently uncovered regulation of ChREBP by cytosolic NADH/NAD^+^ [94,95] operating via elevation in glycerol-3-phosphate levels. This metabolite, which accumulates in CD human carriers and in CD mouse models [34,96], directly binds to ChREBP to activate the DNL transcriptional program. Although promising, further research is required to confirm the participation of this route in the CD phenotype.

Overall, current evidence supports a model in which redox imbalance and metabolite accumulation in CD alter both metabolic and transcriptional regulation of lipid pathways, promoting lipogenesis and potentially impairing FAO, thereby contributing to hepatic steatosis.

## 4. Mechanisms of Ureagenesis Impairment in CD

Protein catabolism requires detoxification of ammonium in the urea cycle (UC) with energy consumption (Figure 3). This pathway occurs in mitochondria and cytosol, requiring coordination of five enzymes, mitochondrial carriers to connect the two compartments, and an accessory enzyme producing the required allosteric activator N-acetyl-glutamate (NAG) (Figure 3) [65,97,98]. Although other tissues, such as gut and kidney tissues, express UC enzymes, the cycle is not complete, producing net arginine synthesis, while ammonium detoxification as urea is only hepatic [99]. UC activity is regulated by substrate and N-acetyl-glutamate availability and post-translational modifications of several enzymes [100]. Mutations in UC enzymes or carriers cause urea cycle disorders (UCDs), characterized by impaired ureagenesis, accumulation of metabolites, and hyperammonemia (HA) [97], although the plasma amino acid profile differs between UCDs, reflecting the affected step.

Thus, elevated citrulline in plasma is associated with abnormal function of mutated ASS1, classified as Citrullinemia type I. However, in the 80s, a patient with citrullinemia showed low hepatic ASS1 activity with unchanged kinetic parameters and normal mRNA and extrahepatic expression of the enzyme, pointing to a secondary cause [4]. The new condition was termed late-onset Citrullinemia type II (presently named AACD). In 1999, citrin was identified as the molecular entity implicated in the disease [2], prompting the characterization of AACD and the consideration of citrin as a second carrier in the cycle [65]. Urea cycle dysfunction in CD likely arises from the integration of multiple factors, including (i) insufficient cytosolic aspartate supply via AGC and redox imbalance, (ii) altered energy metabolism, and (iii) dysregulation of ureagenesis.

### 4.1. Aspartate Limitation: The Classical Hypothesis Revisited

The mitochondrial matrix is the main source of aspartate, since in blood its concentration is among the lowest of all circulating amino acids and below cytosolic levels [101]. In fact, aspartate is the amino acid with the highest levels in mitochondria [101], where it is formed from OAA and glutamate thanks to GOT2 activity. Aspartate synthesized in the mitochondrial matrix is exported to the cytosol primarily through AGCs, affecting cytosolic availability of the amino acid. Saheki et al. proposed that ASS1 flux in UC would be limited by a decreased availability of its substrate, aspartate. Additionally, levels of the enzyme were decreased by an unknown mechanism. Either reduced flux due to limited substrate, lower enzyme levels or a combination of both might contribute to citrulline accumulation in the cytosol [82,102]. However, the enzyme is not consistently decreased across patients [49], and plasma aspartate concentration remains in the normal range in patients across stages [3,42,50,103]. Accordingly, no changes in ASS1 levels were found in either citrin KO or citrin/gpd2 KO mice [24]. However, compromised urea production upon glycine/sucrose administration [54] was observed, consistent with reduced aspartate levels observed in the liver [33] but not plasma [24] of citrin/gpd2 KO mice. Furthermore, oral aspartate can partially normalize ammonia levels, highlighting the central role of limited aspartate supply in CD [54].

Alterations in redox balance are expected to exacerbate aspartate limitation and further impair UC function in CD. Metabolic challenges that increase cytosolic redox imbalance, such as sucrose administration, exacerbate HA in citrin/gpd2 KO mice, whereas in citrin KO mice HA is induced only after combined sucrose and glycine administration, but not by sucrose alone [24,54] (Table 1). Alanine supplementation reverses HA in CD mouse models, likely by generating cytosolic pyruvate, which may partially restore the cytosolic NADH/NAD^+^ balance. Authors hypothesize that this would in turn help increase the OAA availability needed for cytosolic aspartate synthesis by GOT1 reversal [54], as suggested also by a study of ETC inhibition [104], which limits mitochondrial aspartate generation [17]. However, GOT1 reversal would be limited under elevated cytosolic NADH/NAD^+^ ratios, which favor the use of OAA by MDH1, and hence this mechanism for cytosolic aspartate synthesis remains highly hypothetical in a situation of MAS block unless the elevated NADH/NAD^+^ ratio can be restored. Hence, CD causes a limitation of mitochondrial aspartate efflux, reducing cytosolic availability, while the extent to which compensatory cytosolic aspartate synthesis contributes remains uncertain. Whether this limitation is involved in the decline in ASS1 levels remains to be established.

### 4.2. Mitochondrial Support of Ureagenesis: TCA and Energy Metabolism

UC is an energy-consuming pathway and depends on mitochondrial oxidative metabolism. The reactions catalyzed by CPS1 and ASS1 consume ATP [97], so decreased ATP production due to restricted glycolysis (Section 2.1) or FAO (Section 3.2) might negatively impact urea production in CD, independent of aspartate availability. However, direct measurements of hepatic ATP levels or energy charge linked to ureagenesis in the context of CD are currently lacking. In addition, the UC is functionally connected to the TCA cycle through the fumarate or aspartate–argininosuccinate shunt [105], also known as the Krebs bicycle (Figure 3). This shunt recycles fumarate produced by ASL in the cytosol to the mitochondrial matrix to support the TCA cycle and energy production or to serve as a precursor for aspartate [65,106]. Indeed, loss of fumarate recycling in a cytosolic fumarase knockout model leads to reduced aspartate levels and accumulation of argininosuccinate and citrulline in the kidney [107]. Due to this integration, imbalanced flux through TCA (see Section 2.1) might also affect ureagenesis.

In addition, production of obligatory allosteric regulator NAG depends on acetyl-CoA availability, leading to HA in FAO disorders [108,109]. No data are available for this metabolite in published literature using CD models or patient clinical results. However, given the impairment in glycolysis and FAO pathways (see Section 2.1 and Section 3.2), acetyl-CoA levels could become limiting for NAG synthesis. It is tempting to hypothesize that MCT supplementation may provide a source of acetyl-CoA, as a plausible mechanism contributing to the beneficial effects of the dietary supplement [31,91]. However, this mechanism remains speculative, requires in vivo validation, and does not exclude the contribution of other metabolic effects of MCT supplementation.

### 4.3. Post-Translational Mechanisms Linking NAD^+^ Metabolism and the Urea Cycle

UC is regulated through post-translational modifications (PTMs) such as demalonylation, desuccinylation or deacetylation catalyzed by NAD^+^-dependent sirtuins (SIRT) [100]. In particular, SIRT5, which is located both in the mitochondrial and cytosolic compartments [110], is essential for the adaptive increase in ureagenesis during fasting acting on CPS1 [111]. Recently identified lysine sites of desuccinylation in ASS1 by SIRT5 are associated with changes in thermal stability and enzymatic activity [112]. Because sirtuin activity depends on cellular NAD^+^ availability, alterations in NAD^+^ metabolism may influence UC function. In CD, reduced levels of kynurenine, a key intermediate contributing to NAD^+^ synthesis, are observed in post-NICCD patients [76], raising the possibility of altered NAD^+^ metabolism, which remains to be established. Direct evidence linking NAD^+^ metabolism, SIRT5 activity, and reduced ureagenesis in CD is currently lacking, and the contribution of altered PTMs remains highly speculative.

## 5. Open Questions and Future Perspectives

Despite substantial advances in understanding the biochemical consequences of loss of citrin function, several fundamental aspects of the disease remain unresolved. In particular, the mechanisms governing disease progression, the marked male predominance in adult patients, and the possibility of systemic metabolic adaptations remain poorly understood.

CD has been described as a perplexing disorder [55]. After presenting during infancy, the condition resolves and remains clinically silent in many patients, with only a subset of patients progressing to the severe adult manifestation for reasons that remain unknown, with a variable phenotype across patients [1]. This variable clinical course strongly suggests the existence of metabolic adaptations that preserve hepatic homeostasis for years but ultimately fail in a subset of patients. For example, the citrullinemia and mild hyperammonemia observed in NICCD normalize during the compensated stage, whereas both traits re-emerge in AACD [1,3,5]. Moreover, the plasma amino acid profile differs markedly among NICCD, the compensated stage, and AACD [42,50], suggesting that nitrogen metabolism is dynamically remodeled during disease progression. Understanding how nitrogen homeostasis is maintained during the compensated stage and why this adaptation eventually fails in a subset of patients may provide important insight into disease progression and the transition to the adult phenotype. Beyond amino acid metabolism, alterations in carbon partitioning may also contribute to metabolic adaptation. The reduction in glycolytic flux and accumulation of upper intermediates such as glucose-6-phosphate might also affect glycogen metabolism and the pentose phosphate pathway. This metabolite sits at the crossroads of glucose fate, switching from glycolysis and ATP production to storage or NADPH generation by the pentose phosphate pathway [113]. Non-physiological accumulation of glucose-6-phosphate might alter the normal equilibrium between these routes. Interestingly, increased flux through the pentose phosphate pathway and increased NADPH might contribute to lipid synthesis [114], aggravating the steatosis.

A second unresolved question is the marked male predominance in AACD, which is absent in NICCD [1,5,115]. This increased penetrance of AACD in males has been associated with dietary differences between men and women, with increased carbohydrate and alcohol intake reported in men [32]. In addition, differences in pubertal growth have been linked to sex-dependent regulation of lipid metabolism [116]. No sex differences have been reported in citrin or citrin/gpd2 KO mice, and the relevance of sex in disease progression remains unsolved. The liver exhibits high sexual dimorphism, due to the direct action of sex hormones on corresponding receptors in the liver or indirectly by modulation of growth hormone (GH) pattern, which affects in turn gene expression in the liver [117]. As liver metabolism is highly influenced by sex hormones, compensatory changes may differ in males versus females, potentially contributing to differences in phenotype and disease progression, with important implications for the clinical management of CD.

Finally, a systemic approach to the condition is missing. CD is considered a liver-centered metabolic disorder, a view supported by the ability of liver transplantation to permanently correct clinical symptoms [1,4]. However, the liver is increasingly recognized as a central regulator of whole-body physiology, communicating with distant organs through metabolites, hormones, cytokines, bile acids, and neural pathways. Studies in chronic liver diseases have shown that disruption of these signaling networks contributes to dysfunction in multiple organs, particularly the brain, skeletal muscle, and gastrointestinal tract [118,119,120].

Whether systemic alterations occur in CD remains largely unexplored. However, several observations suggest that systemic consequences of hepatic dysfunction may extend beyond the metabolic pathways traditionally studied in CD. Neurological manifestations are a defining feature of AACD [1], mainly associated with HA, showing a direct effect on brain functionality. Moreover, a recent follow-up study of NICCD patients reported increased prevalence of attention deficit and hyperactivity symptoms associated with the severity of neonatal HA [52], opening the possibility of a more subtle effect. In addition, patient-reported outcomes indicate that the compensated stage is not completely asymptomatic, with chronic fatigue and reduced quality of life frequently reported even outside HA crisis [8]. Fatigue, which can be central or peripheral [121], may also reflect the liver dysfunction effect on both brain and muscle, as described in chronic liver disease [121,122]. These observations are to be interpreted cautiously, as the available clinical and patient-reported data do not establish a direct causal link between hepatic metabolic redox alterations in CD and fatigue, carbohydrate aversion, or neurological and attention-related symptoms. Alternative explanations and the limitations of observational and patient-reported measures should therefore be considered, and the relative contribution of hepatic dysfunction to these extrahepatic manifestations remains to be established. Understanding how CD affects systemic metabolic communication may therefore represent an important future research direction and reveal previously unrecognized consequences of chronic hepatic redox imbalance and metabolic adaptation. Investigating interorgan metabolic interactions during the compensated stage may provide insight into disease progression and help identify biomarkers or therapeutic targets before the onset of severe adult manifestations.

## 6. Conclusions

CD is a complex metabolic disorder in which disruption of the MAS impairs hepatic redox balance, leading to widespread alterations in carbohydrate, lipid, and nitrogen metabolism. Accumulating evidence supports a central role for cytosolic NADH/NAD^+^ imbalance in linking metabolic defects to clinical manifestations, including hypoglycemia, steatosis, and hyperammonemia. However, the heterogeneity of disease presentation, the mechanisms governing progression from neonatal to adult forms, and the contribution of systemic metabolic adaptations remain incompletely understood. A deeper understanding of these processes will require integrative approaches combining metabolic flux analysis, longitudinal patient studies, and investigation of interorgan communication. Such efforts could refine the current pathophysiological model of CD and may ultimately guide the development of more targeted and effective therapeutic strategies.

## Figures and Tables

**Figure 1:**
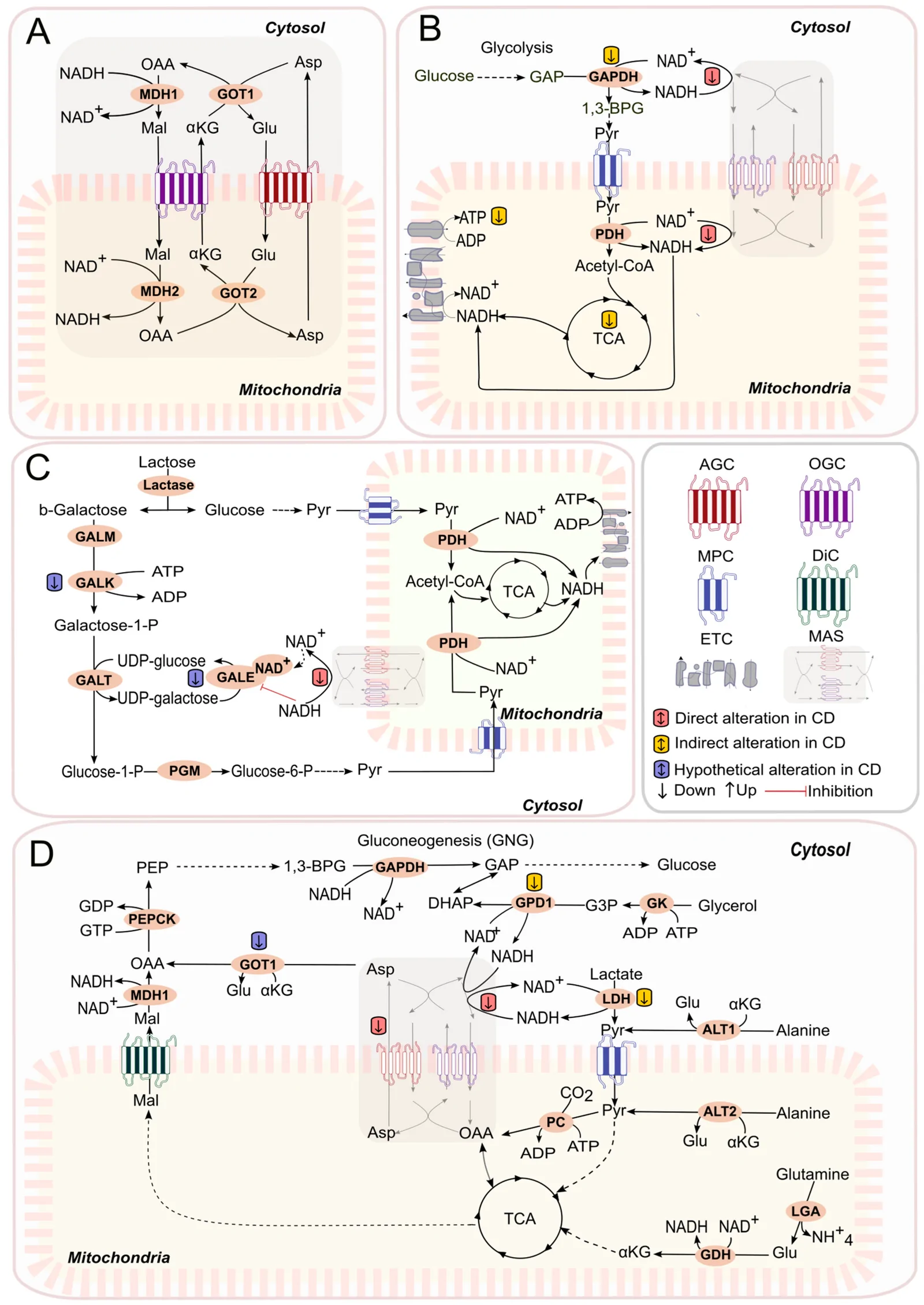
Effects of Citrin Deficiency on carbohydrate metabolism. (**A**) Schematic representation of the malate–aspartate shuttle (MAS) to transfer cytosolic redox equivalents to the mitochondrial matrix. NADH is oxidized to NAD^+^ by cytosolic malate dehydrogenase (MDH1, EC 1.1.1.37) to malate. Malate (Mal) is transported into mitochondria in exchange for α-ketoglutarate (αKG) through the oxoglutarate carrier (OGC/SLC25A11). In the mitochondrial matrix, mitochondrial malate dehydrogenase (MDH2, EC 1.1.1.37) catalyzes the reverse reaction, oxidizing malate to OAA while reducing NAD^+^ to NADH. Transamination of glutamate to oxaloacetate (OAA) by mitochondrial aspartate aminotransferase (GOT2, EC 2.6.1.1) generates αKG and aspartate. Aspartate (Asp) is exported in exchange for cytosolic glutamate plus H^+^ by aspartate/glutamate carriers (AGCs), and the opposite transamination reaction is performed by the cytosolic GOT1 enzyme, producing OAA and closing the cycle. (**B**–**D**) Functional coupling between MAS and carbohydrate metabolic pathways. Alterations are classified as direct (immediate consequences of citrin loss), indirect (downstream consequences supported by experimental observations), or hypothetical (proposed mechanisms not directly demonstrated in CD). Direction of alteration is indicated by arrows (down/up). (**B**) Glycolytic enzyme glyceraldehyde-3-phosphate dehydrogenase (GAPDH, EC 1.2.1.12) activity is affected by elevated cytosolic NADH/NAD^+^ in CD, restricting glycolytic flux toward mitochondrial pyruvate oxidation and ATP production. (**C**) In galactose metabolism, elevated cytosolic NADH/NAD^+^ may compromise UDP-glucose 4′-epimerase (GALE, EC 5.1.3.2) activity; GALK activity is dependent upon ATP availability. (**D**) Redox imbalance affects the utilization of reduced substrates such as lactate or glycerol. Glucose output might also be affected due to impaired efflux of mitochondrial oxaloacetate as aspartate. Dashed lines indicate several steps. 1,3-BPG, 1,3-Bisphosphoglycerate, ALT1/2, alanine aminotransferase cytosolic/mitochondrial; DHAP, dihydroxyacetone phosphate; DiC, dicarboxylate carrier, ETC, electron transport chain, G3P, glycerol-3-phosphate; GALK, galactokinase, GALM, galactose mutarotase, GALT, galactose-1-P-uridylyltransferase, GAP, glyceraldehyde-3-phosphate, GDH, glutamate dehydrogenase; GK, glycerol kinase; GPD1, cytosolic glycerol phosphate dehydrogenase, LGA, liver glutaminase; LDH, lactate dehydrogenase; MPC, mitochondrial pyruvate carrier, PC, pyruvate carboxylase; PEPCK, phosphoenol pyruvate carboxykinase, pyr, pyruvate, PDH, pyruvate dehydrogenase, TCA, tricarboxylic acid cycle.

**Figure 2:**
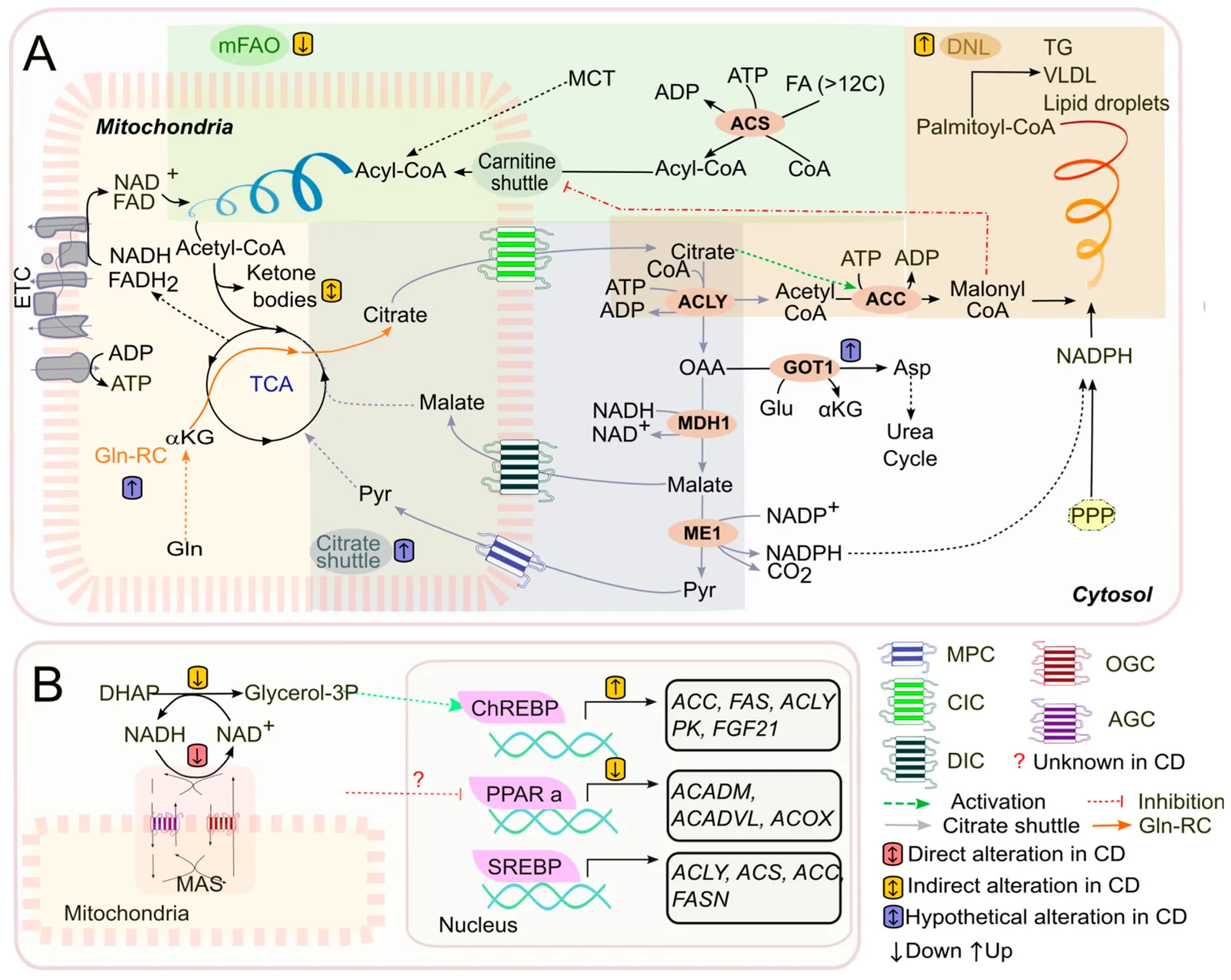
Proposed mechanisms linking mitochondrial redox imbalance to hepatic lipid accumulation in Citrin Deficiency. (**A**) Overview of mitochondrial fatty acid oxidation (mFAO) and de novo lipogenesis (DNL). Hepatic FAO requires activation of FA to Acyl-CoA by acyl-CoA synthetases (thiokinases), consuming ATP. Long-chain fatty acyl-CoA molecules (>12C) require transport into mitochondria through the carnitine shuttle, whereas shorter-chain fatty acids can enter the matrix independently and become acylated in the matrix. In the mitochondrial matrix, acyl-CoA molecules undergo sequential cycles of FAD-linked oxidation, hydration, NAD^+^-linked oxidation and cleavage, with specific enzymes to process short, medium and long molecules. The acetyl-CoA generated can enter the TCA or form ketone bodies. Additionally, NADH and FADH_2_ transfer electrons to the electron transport chain to support ATP synthesis. De novo FA synthesis is catalyzed by the multifunctional enzyme fatty acid synthase (FASN) in a multistep process (condensation, reduction, dehydration, reduction) that consumes NADPH. Successive additions of malonyl-CoA on a growing acyl chain elongate the molecule by two carbons per turn. DNL requires NADPH generated by the pentose phosphate pathway (PPP) or the malic enzyme (ME1). ME1 participates in the citrate shuttle, which also generates acetyl-CoA in the cytosol to support DNL. In CD, hypothetical increased flux through the citrate shuttle might compensate for lack of MAS activity. Citrate exported via the citrate carrier (CIC/SLC25A1) is cleaved by ATP-citrate lyase (ACLY, EC 2.3.3.8) to acetyl-CoA and OAA. Cytosolic OAA is recycled back as malate or as pyruvate through their respective mitochondrial carriers to fuel TCA. This conversion regenerates cytosolic NAD^+^ by MDH1, whereas cytosolic malic enzyme (ME1) generates NADPH. Alternatively, OAA could support cytosolic aspartate production via GOT1, potentially contributing to urea cycle function in the absence of citrin. A hypothetical source of citrate to sustain flux is the reductive carboxylation of glutamine (Gln-RC, orange arrows). (**B**) Proposed transcriptional deregulation of lipid metabolism in CD. Redox-driven accumulation of metabolites such as glycerol-3-phosphate may activate carbohydrate-responsive element-binding protein (ChREBP), promoting lipogenic gene expression. In parallel, downregulation of peroxisome proliferator-activated receptor alpha (PPARα) may limit fatty acid oxidation. SREBP-1 involvement has not been investigated. Alterations are classified as direct (immediate consequences of citrin loss), indirect (downstream consequences supported by experimental observations), or hypothetical (proposed mechanisms not directly demonstrated in CD). Direction of alteration is indicated by arrows (down/up). Pathways are highlighted as shadowed boxes: FAO in green, DNL in brown and citrate shuttle in gray tones.

**Figure 3:**
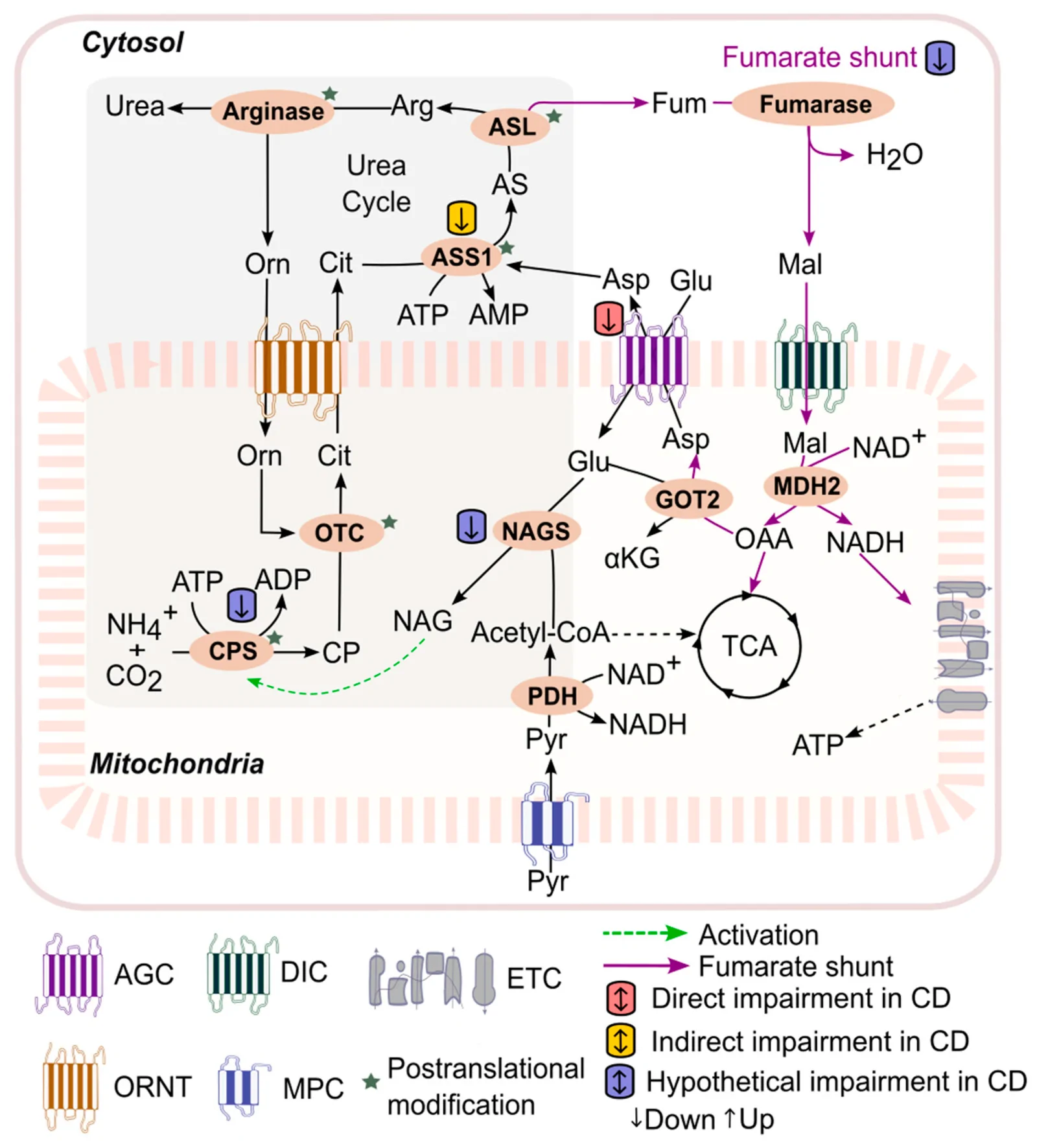
Integration of citrin functions with the urea cycle and mechanisms leading to hyperammonemia. The urea cycle starts with the activity of carbamoyl phosphate synthetase (CPS1, EC 6.3.4.16). CPS1 catalyzes the ATP-dependent formation of carbamoyl phosphate from ammonium and bicarbonate. Then, the enzyme ornithine transcarbamylase (OTC, EC 2.1.3.3) generates citrulline (Cit) from CP and ornithine (Orn) in the mitochondrial compartment. Cit effluxes in exchange for cytosolic Orn through the Orn carrier (ORNT, SLC25A15). In the cytosol, argininosuccinate synthetase (ASS1, EC 6.3.4.5) binds Cit to aspartate (Asp) provided by citrin in a reaction that consumes energy. The successive activities of argininosuccinate lyase (ASL, EC 4.3.2.1) and arginase 1 (ARG1, EC 3.5.3.1) generate urea and Orn, which is recycled back to the mitochondria. The byproduct fumarate (Fum) can be recycled to generate energy in the mitochondrial TCA by the activities of fumarase and the dicarboxylate carrier. N-acetyl-glutamate synthase (NAGS, EC 2.3.1.1) produces in the mitochondria the required allosteric activator of CPS1. Stars indicate enzymes regulated by post-translational modifications depending on sirtuins. Alterations are classified as direct (immediate consequences of citrin loss), indirect (downstream consequences supported by experimental observations), or hypothetical (proposed mechanisms not directly demonstrated in CD).

**Table 1:** Comparative evidence for metabolic alterations in Citrin Deficiency from human, mouse, and cellular studies.

Pathway	Patients	CD Mouse Models ^1^	CD Cellular Models ^2^
Glycolysis	High lactate/pyruvate ratio in plasma as marker of reductive imbalance [29,30,31]. Altered TCA plasma metabolites [29]. CD patients exhibit aversion to carbohydrates [7,32]. Oral glucose tolerance test [31], carbohydrate intake [8] and administration of glycerol worsen clinical symptoms [9]. Fatigue as frequent symptom affecting quality of life [8].	Elevated lactate/pyruvate ratio in perfused livers from citrin KO mice [23] and in livers from citrin/gpd2 KO mice [24]. Elevated hepatic glycerol-3-phosphate in CD mouse models [33,34]. Sucrose administration in CD mouse models decreases TCA cycle metabolites [33] and induces hyperammonemia [24,33]. Citrin/gpd2 KO mice show aversion to alcohol and sucrose [35].	Increased NADH/NAD^+^ ratio in primary mouse hepatocytes [16] and in HepG2 citrin KO cells [26]. HepG2 citrin KO cells show reduced glycolytic capacity and accumulation of upstream metabolites of GAPDH, with decreased downstream metabolites [26]. Decreased TCA intermediates and OXPHOS capacity in HepG2 citrin KO cells [26].
Galactose metabolism (Leloir pathway)	NICCD patients show galactosemia with symptoms such as vomiting and feeding difficulties restored by lactose-restricted diet [36,37]. Galactosemia is linked to hepatotoxicity, intrahepatic cholestasis and cataracts [38,39] that are symptoms reported in NICCD patients [40,41].	No direct evaluation of galactose metabolism in mouse models. Significant increase in ALP in citrin/gpd2 KO mice [24] may indicate cholestatic phenotype.	No direct evaluation of this trait.
Gluconeogenesis	Occurrence of hypoglycemia in CD patients across disease stages, particularly in NICCD and FTTDCD [1,3,5,42,43]. High lactate/pyruvate ratio in plasma as marker of redox imbalance [29,30,31]. Loss of consciousness and fatigue in a catabolic state treated by food or milk intake to manage hypoglycemic symptoms [43].	Hypoglycemia observed in citrin KO and citrin/gpd2 KO mice under fasting, and in citrin/gpd2 KO mice in fed state [24] Perfused liver of citrin KO mice shows decreased glucose production from lactate [23]. Increased TCA cycle intermediates in fasted state consistent with GNG block [33].	No direct gluconeogenic capacity has been measured. Upregulation of gluconeogenic genes: phosphoenolpyruvate carboxykinase 1 and glucose-6-phosphatase in HepG2 citrin KO cells [26].
Lipid metabolism	Fatty liver and/or dyslipidemia reported in NICCD-diagnosed children [5], CD patients classified as FTTDCD [5], and in AACD patients with cirrhosis complications [3]. *PPARα* and FAO genes (*ACADM*, *ACADVL*, *ACOX1*) and corresponding proteins are reduced in patients with steatosis. No changes in fatty acid synthesis genes [44]. Elevated free carnitine and long-chain acyl-carnitines have been reported in CD patients [45,46], suggesting inefficient fatty acid oxidation. Both low [29,47] and elevated [48] plasma ketone bodies indicate deregulation of FAO.	citrin/gpd2 KO mice show increased hepatic triglycerides in fasted animals [24]. Plasma free fatty acids are highly elevated in fasted citrin/gpd2 KO mice [24]. citrin/gpd2 KO mice show increased lipogenic transcription due to increased ChREBP activation [34] that leads to elevated expression of lipogenic genes: *Fasn*, *Acaca*, *Elovl6*, *Acly*, *Agpat2* [24,34].	PPARα is downregulated in CD-HLCs in both low-and high-glucose conditions, and FAO genes (*ACADM*, *CPT1A*, and *FABP1*, but not *ACADVL*) were differentially downregulated in low glucose [27]. HepG2 citrin KO cells show downregulation of FAO genes (*CPT1A/B*, *ACADM*, *ACADVL*, *ACOX1*) and upregulation of the lipogenic gene *FASN* in the studied conditions [26]. β-oxidation is impaired in CD-HLCs [27] and HepG2 citrin KO cells [26].
Urea cycle	Hyperammonemia is a main symptom of AACD patients, also present in a subset of NICCD cases [1,3,42]. Decreased hepatic ASS1 activity aligns with severity [4] but it is not consistently reported across CD patients [49]. Altered amino acid profile differs among presentations [1,36,50,51,52,53], elevated citrulline in NICCD and AACD as main biomarker.	citrin/gpd2 KO mice show increased ammonium levels [24]. Sucrose and glycine/sucrose oral administration induce hyperammonemia, restored by alanine/ornithine administration in citrin/gpd2 KO mice [54]. Elevated plasma citrulline in citrin/gpd2 KO mice [24]. Normal ASS1 activity [24] and reduced hepatic aspartate [33] in CD mouse models.	CD-HLCs show decreased ammonium clearance and decreased ASS1 expression [28]. Increased ammonium in cell medium and lysate of HepG2 citrin KO [26]. Patient-derived fibroblasts show increased mRNA but low levels of ASS1 protein [55].

^1^ Mouse models were generated by global knockout of *Slc25a13* (citrin KO mouse model) [23], showing a mild phenotype that was worsened when combined with global elimination of the alternative NADH glycerol phosphate shuttle in the double citrin/mitochondrial glycerol-3-phosphate dehydrogenase (citrin/gpd2) KO model [24]. ^2^ Cellular models have been generated either from patient-derived fibroblasts or peripheral blood mononuclear cells, which are differentiated into hepatocyte-like cells (HLC), or by CRISPR/Cas9-mediated knockout of *SLC25A13* in HepG2 cells (HepG2 citrin KO cells) [26,27,28].

**Table 2:** Current mechanisms for metabolic alterations in CD.

	Mechanism	Evidence State in CD	Comments
Glycolysis	NADH/NAD^+^ blocks GAPDH	Demonstrated in an experimental model; supported by indirect biochemical evidence in patients	Direct determination of glycolysis intermediates, mitochondrial respiration and NADH/NAD ratios in cell model [26], consistent with indirect determination of plasma lactate/pyruvate in patients [29,30,31].
Galactose	NADH inhibits GALE	Hypothesis	In vitro assay of purified enzyme shows inhibition [56], no data with CD models.
	Impaired glycolysis may limit ATP availability for Leloir pathway	Hypothesis	Biochemically plausible, but no data available from CD models.
Gluconeogenesis	NADH/NAD^+^ limits use of reduced substrates	Demonstrated in an experimental model; supported by indirect biochemical evidence in patients	Observed lower glucose output from reduced precursors from perfused CD mouse livers [23] aligns with hypoglycemia presented by mouse models and patients.
	AGC-dependent efflux as aspartate	Hypothesis	Biochemically plausible, supported by historical biochemical description and data in other cell model [57]. No evidence in CD models or patients.
Lipid metabolism	Citrate shuttle increase	Hypothesis	Proposed compensatory mechanism to restore redox balance and cytosolic aspartate levels, supported by indirect molecular evidence (increased *Acly* in CD mouse model [24]), not directly evaluated in CD patients, mouse or cellular models.
	PPARα downregulation reduces FAO-enzymes	Demonstrated in a subset of patients, demonstrated in cell models	Direct determination of PPARα and targets in AACD patients [26]. Requires validation in other stages. FAO was decreased in cell models [26], requires validation of FAO flux in vivo.
	Glycerol-3-phosphate regulation of ChREBP	Demonstrated in experimental model, supported by indirect molecular evidence	ChREBP activation is demonstrated in CD mouse models by increased ChREBPβ and target-gene expression. In humans, it is supported by elevation of downstream targets in CD heterozygous carriers [34], requires validation.
Urea cycle	Aspartate availability limits ASS1 reaction	Supported by indirect evidence	Decreased hepatic aspartate in CD mouse models [33], and decreased brain aspartate in aralar knockout models [13]. Importance of aspartate supply is supported by observation such as oral aspartate can partially normalize ammonium levels [54]
	Decreased ASS1 levels block UC	Demonstrated in a subset of patients	Direct determination of hepatic ASS1 levels in a subset of AACD patients. Decrease is not consistent across CD stages/patients [49]. Not reproduced in CD models.
	Imbalance flux through TCA, NAG synthesis and PTM may alter UC function in CD	Hypothesis	No direct data in CD, currently speculative. New proposed mechanisms based on known relevance of UC-TCA connection, role of NAG in regulation of CPS1 activity and NAD+-dependent Sirtuins regulation of urea cycle enzymes.

## Data Availability

No new data were created or analyzed in this study. Data sharing is not applicable to this article.

## Abbreviations

AACD	Adolescent and adult Citrin Deficiency
ACC	Acetyl CoA carboxylase
ACLY	ATP Citrate lyase
AGC	Aspartate/glutamate carrier
ASL	Argininosuccinate lyase
ASS1	Argininosuccinate synthetase 1
CD	Citrin Deficiency
ChREBP	Carbohydrate response element-binding protein
CPS	Carbamoyl phosphate synthetase
CPT1	Carnitine palmitoyl transferase 1
CTLN2	Citrullinemia type II (now termed AACD)
DNL	De novo lipogenesis
FA	Fatty acid
FAO	Fatty acid oxidation
FASN	Fatty acid synthase
FTTDCD	Failure to Thrive and Dyslipidemia caused by Citrin Deficiency
GALE	UDP-glucose 4′-epimerase
GALK	Galactose kinase
GALM	Galactose mutarotase
GALT	Galactose-1-phosphate uridylyltransferase
GNG	Gluconeogenesis
GOT1	Cytosolic glutamate/oxaloacetate transaminase/aspartate transaminase
GOT2	Mitochondrial glutamate/oxaloacetate transaminase/aspartate transaminase
Gpd2	Glycerol-3-phosphate dehydrogenase, mitochondrial
HA	Hyperammonemia
HLC	Hepatocyte-like cells
MAS	Malate–aspartate shuttle
MCT	Medium chain triglycerides
NAGS	N-Acetyl glutamate synthase
NICCD	Neonatal Intrahepatic Cholestasis caused by Citrin Deficiency
OAA	Oxaloacetate
ORNT	Ornithine mitochondrial transporter
OTC	Ornithine transcarbamylase
OXPHOS	Oxidative phosphorylation
PPARα	Peroxisome Proliferator-Activated Receptor alpha
PTM	Post-translational modification
SIRT	Sirtuin
SREBP	Sterol response element-binding protein
TCA	Tricarboxylic acid cycle
TG	Triglyceride
UC	Urea cycle
UCD	Urea cycle disorders
VLDL	Very-low-density lipoprotein
